# Using system dynamics to assess the complexity of rural toilet retrofitting: Case study in eastern China

**DOI:** 10.1016/j.jenvman.2020.111655

**Published:** 2021-02-15

**Authors:** Yong Li, Shikun Cheng, Zhengyi Li, Hongqing Song, Miao Guo, Zifu Li, Heinz-Peter Mang, Yuning Xu, Cong Chen, Davaa Basandorj, Lingling Zhang, Tianxin Li

**Affiliations:** a School of Energy and Environmental Engineering, Beijing Key Laboratory of Resource-oriented Treatment of Industrial Pollutants, University of Science and Technology Beijing, Xueyuan Road No.30, Haidian District, Beijing, 100083, PR China; b School of Civil and Resource Engineering, University of Science and Technology Beijing, Xueyuan Road No.30, Haidian District, Beijing, 100083, PR China; c Department of Engineering, Faculty of Natural & Mathematical Sciences, King's College London. Strand Building S2.22, London, WC2R 2LS, United Kingdom; d German Toilet Organization, Paulsenstr. 23/12163 Berlin, Germany; e Jiaozhou Committee for Patriotic Sanitation Campaign, Beijing Road No.61, Jiaozhou, Qingdao, 266300, PR China; f School of Economics and Management, University of Science and Technology Beijing, Xueyuan Road No.30, Haidian District, Beijing, 100083, PR China; g Water Research Center, Mongolian University of Science and Technology, Baga toiruu, Khoroo No.8 Sukhbaatar District, Ulaanbaatar, 14191, Mongolia

**Keywords:** Toilet retrofitting, Fecal sludge, Rural sanitation, System dynamics, Toilet revolution

## Abstract

Rural toilet retrofitting (RTR) is a complex, dynamic system that is affected by many factors and the positive/negative feedback relationships between subsystems and variables. Traditional technologies and management methods face challenges in fundamentally describing and solving problems in RTR. To bridge this gap, this study utilizes system dynamics and causal loop diagrams to explain such problems based on data collected from the stakeholders of the RTR in Jiaozhou from 2018 to 2019. Specifically, this study examines the RTR system from the perspectives of household users, wastewater treatment plants, local governments, grassroots promoters, operation and maintenance personnel, toilet supplier and construction teams, and fecal sludge end users. The factors and processes involved in RTR are identified, and the feedback and relationships among its major stakeholders are established. Results show that the motivation of farmers to engage in RTR is a key variable that affects their final decisions regarding retrofitting and maintaining toilet functions. Meanwhile, the important variables related to the feedback and relationships among the major stakeholders of RTR are mostly focused on policies, subsidies, technology, satisfaction, and cooperation. A scientific analysis method and the updated RTR plan for toilet revolution are then formulated to promote the implementation of RTR in developing countries.

## Highlights

• Typical case of rural toilet retrofitting in China is selected and investigated.
• System dynamics model for simulating rural toilet retrofitting is developed.
• Causal loop diagrams for rural toilet retrofitting are presented and analyzed.
• Experience of rural toilet retrofitting is shared for other developing countries.

## Author contribution

Yong Li: Writing - original draft, Visualization, Shikun Cheng: Conceptualization, Methodology, Writing - review & editing, Supervision, Zhengyi Li: Investigation, Data curation, Software, Hongqing Song: Investigation, Data curation, Software, Miao Guo: Software, Writing - review & editing, Zifu Li: Methodology, Writing - review & editing, Heinz-Peter Mang: Writing - review & editing, Yuning Xu: Resources, Tianxin Li: Funding acquisition, Supervision.

## 1 Introduction

A total of 1.5 billion people around the world lack access to basic sanitation services (WHO, 2019), and only two-fifths of the population in rural areas have access to safe sanitation facilities (Weststrate et al., 2019). Meanwhile, 673 million people around the world continue to practice open defecation (UNICEF and WHO, 2019). The poor sanitation infrastructure in rural settlements contribute to the unsafe disposal of feces and urine resources, especially in developing countries (Rashid and Pandit, 2017).

Human feces and urine are rich in nutrients and highlight the potential of converting pollutants into valuable resources (Shi et al., 2018). However, when feces are not completely removed from the human living environment, they can come in direct or indirect contact with people and cause acute gastrointestinal diseases (Delahoy et al., 2018), especially among children (Bawankule et al., 2017) and immunocompromised pregnant women (Magri et al., 2015; Nyambe et al., 2020; Penakalapati et al., 2017). The sanitation infrastructure in rural areas is relatively backward compared with that in urban areas (Zhang et al., 2020). For instance, in developing countries, feces cannot be safely stored in time due to the lack of necessary sanitation facilities (Kwiringira et al., 2014). Moreover, rural people are exposed to a significantly higher risk of suffering from diseases owing to the poor sanitation infrastructure in their areas compared with their urban counterparts (Snehalatha and Anitha, 2012). Improving sanitation in rural areas has been highlighted as a global objective (Murungi and Dijk, 2014), and many countries, in response, have implemented a series of policies and plans to increase the number of sanitary toilets and improve their rural sanitation infrastructure to eliminate the random discharge of rural fecal sludge (FS) (Penn et al., 2018; Rashid and Pandit, 2017).

The Chinese government promotes a rural “toilet revolution” (Zhang et al., 2020) that aims to increase the prevalence of sanitary toilets to 85% and 100% by 2020 and 2030, respectively. However, the achievement of such goal is hindered by several factors. First, an imbalanced toilet coverage is observed between rich and poor areas. Second, some of the installed toilets are unable to operate in the absence of a sewage system or cannot be used in winter due to the severe cold temperature and water shortage during this period. Third, the construction of millions of toilets within a short period is mainly subsided by the government (Chen, 2010; Roubík et al., 2020), and without such subsidy, the construction would impose a heavy financial burden on farmers.

In October 2014, India launched a similar program called Swachh Bharat Mission (SBM) (Biswas et al., 2020; Caruso et al., 2019; Rashid and Pandit, 2019), which aims to eliminate open defecation in rural areas in the next 5 years. However, eliminating open defecation in India remains a challenge. A survey of 3235 families and 22,787 respondents from 5 Indian states in 2014 revealed that more than 40% of families with working latrines have at least one member still defecating in the open (Coffey et al., 2014). In addition, a follow-up study in late 2018 revealed that about 40%–50% of the rural population in these states continued to defecate in the open. Nevertheless, this percentage is much lower than that recorded in the 2014 survey (about 70%). These findings contradict the claim of SBM that open defecation has been completely or largely eliminated (Gupta et al., 2019). Researchers in India have identified multiple factors associated with the persistence of open defecation in the country, such as lack of high-quality toilets with good drainage, rapid filling of toilet pits, large size of households, gender, age, livelihood, and preferences of users, remote areas, and social ability of users during open defecation (Caruso et al., 2019; O'Reilly et al., 2017; Routray et al., 2015).

Sustainable toilet systems involve an ecological chain that revolves around human waste collection, storage, transportation, treatment, disposal, and utilization (Ding et al., 2012). Rural “toilet revolution” aims to promote a sustainable toilet system by building sanitary facilities that achieve a safe and sanitary separation of people and human excreta and utilize technical approaches for resource recovery (Cheng et al., 2018; Shen et al., 2018). A sustainable rural toilet system should be investigated in depth, and optimal toilet retrofitting models should be built.

This study examines the sustainable toilet system that was built as a result of the rural toilet retrofitting (RTR) in Jiaozhou, Shandong Province, located in Eastern China. Qualitative system dynamics (i.e., causal loop diagram or CLD) are used to investigate the endogenous feedback relationships among the stakeholders in RTR (Cavicchi, 2016; Roubík et al., 2020). Specifically, CLD maps the causal processes of RTR in Jiaozhou by using information gathered from semi-structured interviews, public documents, and publications relevant to the case study (Liu et al., 2019b). However, to date, no study has applied system dynamics to investigate RTR, and only few studies have systematically analyzed the stakeholders in RTR, including household users, wastewater treatment plants (WWTP), local governments, grassroots promoters, operations and management (O&M) personnel, toilet supplier and construction teams, and FS end users. This paper aims to identify all factors in RTR and formulate better RTR policies and action plans.

## 2 Materials and methods

### 2.1 Theory and methodology

System dynamics is a methodology for studying non-linearity, information feedback, and dynamic complexity systems (Jiang et al., 2020; Richardson, 2011). This concept was introduced by the late J. W. Forrester in 1958, which marked the first time for system dynamics to be applied in industrial research (Forrester, 1997; Papachristos, 2019). The basic principles and methods of system dynamics were introduced in 1968 (Papachristos, 2019), including determining the variables existing in systems, analyzing the causality among these variables from a dynamic perspective (Roubík et al., 2020), establishing CLDs, stock and flow diagrams, and equations of systems that represent the relationships among variables, and structurally simulating real systems (Swanson, 2002). System dynamics generally aims to optimize the parameters, structures, and boundaries of system structures (Wang et al., 2018). The executor can make reasonable choices from the large amount of data and information obtained and then transform these information resources into continuous decisions (Liu et al., 2019a). An effective strategy has also been designed to examine several variables and their interconnections with real problems (Mutingi et al., 2017).

System dynamics has also been applied to investigate various complex systems in ecology, economics, management, and other fields (Honti et al., 2019). By comparing different scenarios, researchers can focus on the development of a single discrete event and conduct a behavioral analysis of the overall development dynamics to improve their understanding of complex systems (Forrester, 1994). Given that feedback characteristics indicate that the internal components of systems are causal to one another, the feedback closed loop of the system dynamics model must contain all variables related to the internal structure of the system instead of using external or random related variables (Forrester, 1961). The system dynamics simulation model expresses the examined object as a system of first-order differential equations (Roubík et al., 2020).

**Fig. 1:**
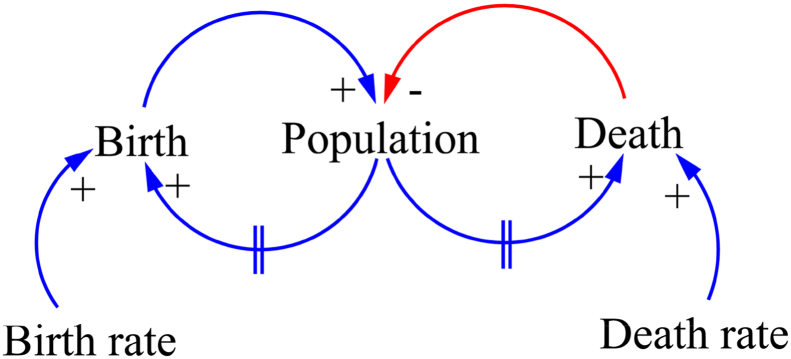
Example of CLD

To ensure the validity and reliability of qualitatively examining the RTR in Jiaozhou, we initially defined the iterative and reflexive processes of empirical research (Morse, 2001; Noble and Smith, 2015) and then drew our final CLD by using the VENSIM software. We then employed this CLD to evaluate the RTR in Jiaozhou. However, this diagram does not enable a mathematical modeling of the system (Roubík et al., 2020). Table 1 shows the basic building blocks of system dynamics in CLD models as represented by icons. The employed CLD consists of variables that are connected to directional links that denote the impact of variables. Feedback can be either positive (with a “+” sign) or negative (with a “–” sign). Fig. 1 presents an example of CLD (Ma and Wang, 2019). An increase in birth rate corresponds to an increase in the number of births, which subsequently results in an increase in total population, thereby generating positive feedback (“+”). As the population continues to grow, the birth will also increase but with a certain time delay. By contrast, an increase in death rate corresponds to an increase in the number of deaths and a reduction in the total population, thereby showing a negative feedback (“–”). An increase in population may also be accompanied by an increase in the number of deaths but with a certain time delay (Cavicchi, 2016; Ma and Wang, 2019).

**Table 1:** Symbols used in the causal loop diagram.

Symbols	Illustrations	Mathematics
	“If X increases, then Y increases above the original value.” “In the case of accumulation, X adds to Y” (Roubík et al., 2020).	$\frac{\delta y}{\delta x}>0$ In the case of accumulations, $Y=\overset{T}{\underset{T_{0}}{\int }}\left(X+\ldots \right)dt+Y_{T_{0}}$
	“If X increases, then Y decreases below the original value.” “In the case of accumulation, X is subtracted from Y” (Roubík et al., 2020).	$\frac{\delta y}{\delta x}<0$ In the case of accumulations, $Y=\overset{T}{\underset{T_{0}}{\int }}\left(-X+\ldots \right)dt+Y_{T_{0}}$
	Delay mark

In this study, CLD represents the output of all aggregated data, including the information collected from semi-structured interviews, statistical yearbooks, reports, and publications related to the case study (Liu et al., 2019b; Qingdao Municipal Statistics Bureau, 2019). As our main data collection method, semi-structured interviews were conducted between 2018 and 2019, and the respondents were recruited via random sampling. However, given that the information provided by random interviewees may be partially biased, such information was integrated and cross-checked with related materials, such as data from statistical yearbooks, reports, publications, and key providers.

CLDs are used to investigate different scenarios and demonstrate the behavior of systems by considering social, economic, and sustainability aspects (Zenezini and Marco, 2020). CLDs can also facilitate issue structuring, organizational learning, and problem solving (Cavicchi, 2016). In this sense, CLDs are suitable for analyzing the structure of RTR in Jiaozhou as a result of the feedback relations among policy documents, perceptions and actions of stakeholders, and ecological elements.

### 2.2 Methods and data sources

**Fig. 2:**
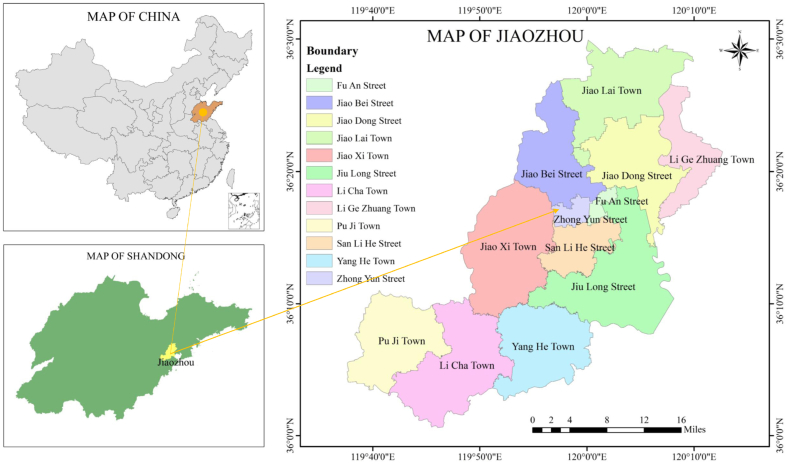
Location of the target areas: Jiaozhou, Shandong Province, China.

A qualitative and systematic analysis was performed to evaluate those factors and processes involved in RTR from the perspectives of household users, WWTPs, local governments, grassroot promoters (e.g., village heads and secretaries), O&M personnel, toilet supplier and construction teams, and FS end users (e.g., gardens and orchards) in the rural areas of Jiaozhou. Jiaozhou is located in an eastern warm temperate semi-humid monsoon region with a land area of 1323.65 km^2^ (Fig. 2). It has an annual average temperature of 13.5 °C and average precipitation of 741.9 mm/year (Qingdao Municipal Statistics Bureau, 2019). Jiaozhou consists of 6 streets, 6 townships, and 811 villages (Liu et al., 2019b). To promote sanitation in its rural areas, the Jiaozhou local government launched its “toilet revolution” program in 2015 and prepared a series of government documents to support this campaign (Liu et al., 2019b). Table 2 presents the three RTR models adopted in this study according to the local water supply and drainage infrastructure conditions in Jiaozhou.

**Table 2:** Three models of RTR in Jiaozhou.

Scenarios	Characteristics
(1) Municipal WWTP with sewer system	Three-chamber septic tank toilets are constructed for each household in villages located near urban areas. FS is transported to WWTPs through the underground pipe network for professional treatment and resource recovery.
(2) Township-level WWTP with local pipeline system	Regional small centralized WWTPs are established among villages located away from urban areas and where a pipe network has been established. Three-chamber septic tank toilets are built for users in the surrounding villages. FS enters WWTP through the underground pipe network for professional treatment and resource recovery.
(3) Village-level decentralized WWTP without pipeline system	The villages are located away from urban areas, towns, industrial clusters. A three-chamber septic tank or double-vault funnel is installed in each household for the preliminary pretreatment of FS liquid. Afterward, the treated FS is transported to the treatment station by truck for professional treatment and resource recovery.

A total of 164 household users participated in the questionnaire survey. In addition, 6 WWTPs and adjacent gardens, 7 members of the leading group of the RTR government, 10 village heads, 8 O&M personnel, and 4 toilet supplier and construction teams were interviewed for approximately 158 h in a semi-structured manner. The collected data were cross-checked with the observations. The raw data were subjected to iterative and reflexive processes, and the CLD was repeatedly inspected and updated until the final version was obtained to achieve a consistent and complex observation of the RTR in Jiaozhou. The information used in this study was collected and processed as follows: (1) the data were collected by conducting a questionnaire survey among household users and by interviewing participants in the RTR; (2) the collected data were cross-checked by conducting in-depth interviews with key information providers; and (3) the data were verified and supplemented with information obtained from the related literature.

The initial CLD was drawn based on the questionnaire survey data, including basic information about RTR policies and subsidies, RTR models, RTR technologies, difficulties faced in RTR, household user satisfaction, relevant actors, and FS management. However, some causal relationships in this initial CLD were inconsistent or incomplete. Therefore, interviews were carried out helped to clarify doubts and to further revise the CLD. The revised CLD was then cross-checked by using data collected from regional statistical yearbooks, related reports, and published books. The data then underwent continuous iteration and repeated cross-checking processes to improve the reliability and validity of the CLD (Cavicchi, 2016; Haraldsson, 2004). Previous studies on the rural toilet revolution provided useful data for justifying the explanations presented in the case study (Cavicchi, 2016; Cheng et al., 2018; Huo, 2019; Xiao, 2019).

## 3 Results and discussion

### 3.1 Revealing the dynamic complexity of RTR through CLD

**Fig. 3:**
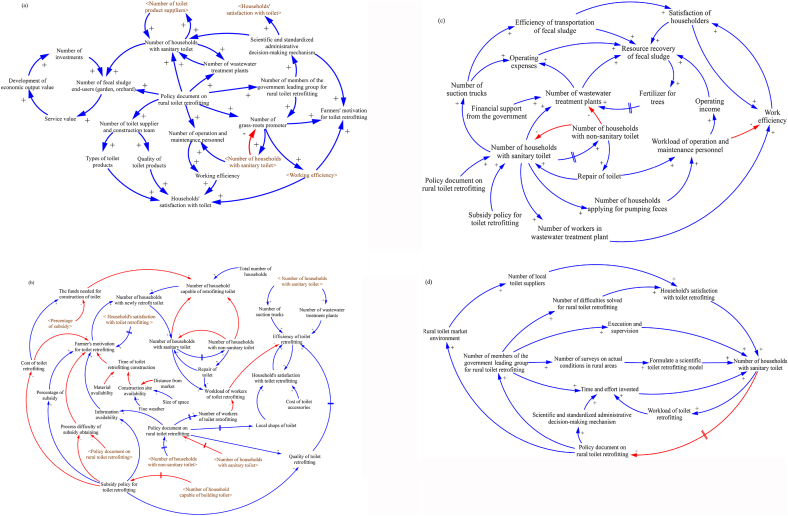

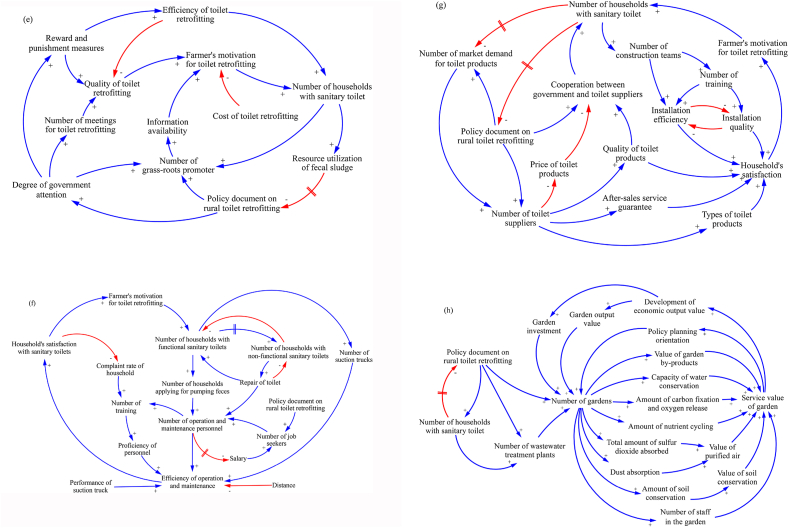
CLD of the participants in RTR in Jiaozhou (a: main CLD of RTR in Jiaozhou; b: from the perspective of household users; c: from the perspective of WWTPs; d: from the perspective of local governments; e: from the perspective of grassroots promoters; f: from the perspective of O&M personnel; g: from the perspective of toilet supplier and construction teams; and h: from the perspective of FS end users).

This section introduces the seven main stakeholders in the process of RTR in Jiaozhou. Fig. 3a shows the main CLD of RTR in Jiaozhou with feedback as closed-loop structures that connect the individual variables. The RTR in Jiaozhou is mainly powered by a related policy document, which increases the number of major stakeholders through positive feedback, construction of WWTPs, and establishment of toilet O&M teams and FS end users. However, the RTR in some provinces and cities only focuses on the number of toilets, rather than on toilet construction quality, toilet O&M, and FS resource recovery due to insufficient funding (Huo, 2019; Xiao, 2019). The vitality of the local rural toilet market cannot be stimulated by implementing RTR and additional toilet suppliers cannot be attracted to the countryside to provide farmers with improved toilet facilities, either.

To ensure the long-term benefits of rural toilet revolution, the Jiaozhou municipal government issued several scientific policy documents, such as the *Implementation Opinions on Establishing a Long-Term Management Mechanism for Rural Toilet Revolution*, *Notice on Establishing a Confirmation Mechanism for the Extraction of Fecal Sludge from Rural Sanitary Toilets*, and *Implementation Opinions on the Acceptance of Rural Toilet Revolution in Jiaozhou* (Liu et al., 2019b). Some researchers argue that through the division of responsibilities, supervision and inspection, assessment, and rewards and punishments and the implementation of specific measures, RTR can be completed and user satisfaction can be subsequently improved by ensuring co-management, close collaboration, and strong promotion (Xiao, 2019).

As shown in Fig. 3a, the growth in the number of sanitary toilets in rural Jiaozhou will decelerate without the robust policy support. Therefore, investments in rural public sanitation facilities, such as WWTPs and public pipeline networks, should be increased. Preferential policies for toilet companies should also be provided, and capable farmers must be encouraged to engage in RTR in order to stimulate the vitality of the rural toilet market.

#### 3.1.1 From the perspective of household users

**Fig. 4:**
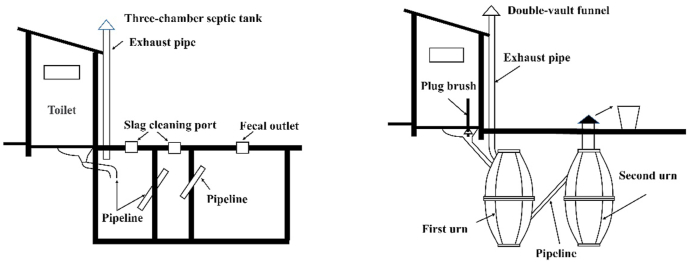
Three-chamber septic tank and double-vault funnel toilet.

Jiaozhou has a population of approximately 900,500 people, 547,900 of whom live in rural areas. The rural area of Jiaozhou houses approximately 89,300 people who are directly involved in farming and related activities, such as wheat, peanut, and vegetable cultivation, forestry, animal husbandry, and fishery (Qingdao Municipal Statistics Bureau, 2019). All interviewed farmers have access to tap water, and some surveyed farmers are involved in off-farming activities, such as small shops and catering. Some of their family members migrate to the city to develop their careers. Meanwhile, the majority of the feces and urine in these areas are collected by using pit latrine before the toilets are retrofitted. Poor sanitation leads to the uncontrolled transmission of parasites and diseases (Zhang et al., 2020), and the current “toilet revolution” requires a widespread construction of improved toilets in rural areas. The survey respondents had varying levels of education, including no education (16.13%), primary education (64.52%), and secondary education (19.35%). Some studies have shown that years of formal education are an important determinant of toilet technology adoption. The majority of the surveyed families have accepted the retrofitted toilet and installed a three-chamber septic tank or double-vault funnel toilet system in their households (Fig. 4) following the national standard *GB 19379-2012 Hygienic Specification for Rural Household Latrine* (Hu et al., 2016; MoH and SAC, 2012). While all investigated villagers were encouraged to participate in RTR training programs, only 77.42% of the survey respondents confirmed that they have participated in such programs. Nevertheless, these respondents rated the current RTR technology in their areas as satisfactory.

CLD was used to examine the retrofitting process and the sustainable outcomes of rural toilets in Jiaozhou from the perspective of household users (Fig. 3b). Subsidy plays a critical role in motivating farmers to engage in RTR. The impact of subsidy policies can be expressed as either stimulation (i.e., percentage of subsidy and information availability) or suppression (i.e., difficulty of obtaining a subsidy). The Jiaozhou municipal government intensified its publicity campaigns and provided sufficient subsidies for RTR to expand the coverage of rural sanitary toilets. The current Jiaozhou RTR subsidy policy provides farmers with subsidies accounting for 90%–100% of the total cost of RTR (1100 CNY^1^) and provides them with squatting pans, water pipes, cement, sand, bricks, steel rods, and other materials for retrofitting. If farmers want to install other facilities, then they need to pay additional costs which are not covered by this subsidy. The government divides administrative villages into units to facilitate the implementation of RTR. While the vast majority of farmers are willing to participate in such activity, in the future, the actual cost of RTR which is borne by farmers will increase after the expiration of the government subsidy policy, thereby requiring them to secure additional funds.

The motivation of farmers to participate in RTR is also affected by technical aspects, such as availability of construction sites. An area of at least 5.5 m^2^ is required to build a qualified three-chamber septic tank, whereas an area of at least 3.8 m^2^ is required to build a double-vault funnel toilet (MoH and SAC, 2012). Another factor that influences such motivation is the availability and accessibility of the necessary building materials, which, in practice, include water pipes, cement, sand, bricks, and steel rods. The distance of remote rural areas from the town should also be considered. Specifically, such distance increases the costs of construction materials and transportation, and thereby hinders the motivation of farmers to participate in RTR. We also consider the impact of weather on the construction and find that non-rainy weather will promote RTR. A higher motivation of households to engage in RTR increases the number of sanitary toilets in the village. However, having sanitary toilets at home is not the same as having functional sanitary toilets. Some users are also occasionally unwilling to use new toilets for various factors, such as clogged water pipes and manure storage tanks filled with FS. Regular maintenance can prevent the failure of toilets (Roubík et al., 2020), and a timely maintenance and pumping ensures the normal usage of these toilets.

The satisfaction of farmers with RTR can also motivate them to engage in RTR, which provides a variety of benefits and is owned by them or by their neighbors. First, the RTR campaign provides every farmer with access to sanitary toilets, which greatly increases the penetration rate of rural sanitary toilets and facilitates a safe collection of FS. However, the abovementioned workload is particularly heavy; for instance, having a limited number of workers will delay the overall progress of RTR (Roubík et al., 2020). Second, the FS needs to be transported by suction trucks and safely processed by centralized treatment stations or WWTPs. By improving the necessary sanitation service facilities, households become willing to participate in RTR and improve its efficiency. Third, along with the continuous retrofitting of rural toilets, the increasingly perfect rural toilet market environment can further motivate farmers to retrofit toilets as reflected in the increased number of local toilet shops, easily obtainable toilet accessories, and high-quality toilet products.

The number of households that are capable of retrofitting toilets also defines the number of households with newly retrofitted toilets and ultimately determines the number of households with three-chamber septic tank or double-vault funnel toilets. In other words, the number of households with sanitary toilets tends to saturate as the RTR progresses.

From the perspective of farmers, solving the problems related to RTR subsidies, RTR technology, and satisfaction with RTR will actively promote the rural toilet revolution.

#### 3.1.2 From the perspective of WWTPs

FS management is composed of emptying, collection, and transport services (from household users to resource recycling, disposal, and safe final use; Schoebitz et al., 2017). To curb the adverse effects of environmental pollution resulting from the uncontrolled discharge of FS, effective measures must be taken to ensure the safe transportation and disposal of FS and to realize FS resource recovery in rural areas (Huo, 2019). The construction and operation of WWTPs aim to ensure the safe treatment of FS in the region. A total of 20 WWTPs, including 1 municipal WWTP with sewer system, 10 township-level WWTPs with local pipeline system, and 9 village-level decentralized WWTPs without pipeline system, were built in Jiaozhou (Liu et al., 2019b). The FS suction truck is equipped with a standard configuration of 1500 people/vehicle. The construction cost of WWTPs and purchase cost of suction trucks are jointly borne by the government and property companies. The normal operation of WWTPs has a positive effect on effective FS resource recovery (Fig. 3c), whereas government financial support plays a decisive role in the construction of WWTPs in rural areas (Benito et al., 2020). Some provinces and cities cannot build WWTPs in their rural areas owing to insufficient funds (Huo, 2019). To increase financing capacity and reduce the risk of incurring local debt, the public–private partnership model is applied in rural WWTP projects (Yang et al., 2017). Researchers find that a win–win situation can be achieved through the cooperation between government public departments and relevant market property companies in providing public products and services (Yang et al., 2017). On one hand, the application of the public–private partnership model in rural FS treatment will allow the government to improve the quality of its services, enhance the processing power and efficiency of rural FS treatment, and cultivate social capital in FS pollution control. On the other hand, property companies can receive investment returns by charging fees for FS treatment, and thereby improve the efficiency of FS resource recovery and service quality (Gianico et al., 2015; Wang et al., 2015).

To guarantee the normal operation of WWTPs, the CLD diagram in Fig. 3c shows that first, increasing the number of households with sanitary toilets will also increase the number of required FS suction trucks and subsequently enhances the efficiency of FS transportation (operating expenses) and FS resource recovery. Second, when sanitary toilets are used for two to four months, the number of households that apply for FS extraction increases (because the storage tank is filled with FS), which is then followed by an increase in the workload of O&M personnel. An increase in the workload of O&M personnel also increases the operating income of WWTPs and eventually increases the efficiency of FS resource recovery, thereby resulting in a positive feedback. Survey results show that users are only charged 25% of the FS extraction fee/time, whereas the remaining 75% of the fee is borne by the government. This charging method can also prevent users from frequently applying for FS extraction and lays the foundation for the market operation of toilets.

The heavy workload of O&M personnel can reduce their work efficiency, and the inability to address toilet failures in time will eventually reduce the satisfaction of households. Therefore, innovating the O&M concepts of WWTPs can increase the efficiency in solving practical problems (Shen et al., 2020). Moreover, recruiting more specialized workers is beneficial to maintaining the normal operation of WWTPs.

#### 3.1.3 From the perspective of local governments

Many researchers believe that the government is of great significance in RTR (Chen and Liu, 2017; Jan and Akram, 2018). In Jiaozhou, the RTR leading group formed by the Jiaozhou municipal government implemented a RTR program that provides the guidelines, requirements, methods, implementation steps, financial security (a RTR subsidy of 1100 CNY per household), and publicity work for RTR (Liu et al., 2019b).

Since 2015, the impact of the RTR on rural human settlements have raised social concerns. In some provinces, such as Zhejiang and Hebei, local governments have established a leading group to guide the scientific implementation of RTR (Liu et al., 2019b). Fig. 3d illustrates how the Jiaozhou government has scientifically implemented RTR to increase the penetration rate of sanitary toilets in rural areas. One key player in this program is the RTR leading group, which disseminates information about policy documents on RTR and coordinates with the involved stakeholders. Researchers from rural areas in China have confirmed that if farmers obtain information about RTR from the government than from other sources, they become likely to retrofit sanitary toilets (Qu et al., 2013). Achieving the political goal indicated in RTR documents is a difficult task and requires the participation of additional government personnel. To scientifically implement RTR, the actual situation in the countryside, including the distribution of villages, housing density, toilet technology selection, geological characteristics, and number of toilets to be retrofitted, should be considered. The impact of the RTR promotion by the government on the number of households with sanitary toilets is represented by four causal loops that vary in stimulating (i.e., number of challenges solved in RTR, execution, and supervision, number of surveys on actual conditions in rural areas, and time and effort invested) the motivation of farmers to retrofit toilets and to contribute to the increase in the number of households with newly built toilets. The currently available technologies for RTR provide farmers with three-chamber septic tank or the double-vault funnel toilets, and the RTR mode shown in Table 2 is adopted for full implementation. If farmers encounter difficulties in the RTR process, then they can receive additional assistance from local governments. However, despite adopting the same subsidy and implementation methods, some villages still fail to complete high-quality RTR, thereby reducing the satisfaction of farmers. Therefore, the members of the government leading group for RTR have to strengthen their execution and supervision capabilities from the beginning of the RTR in order to form a complete toilet system ecological chain. Moreover, the current RTR requires an implementation of a scientific and standardized administrative decision-making mechanism to achieve the goal of the toilet revolution.

A well-established RTR policy influences the rural toilet market environment and subsequently affects the number of high-quality brand toilet suppliers in rural areas, the satisfaction of RTR households, and the number of households with sanitary toilets. Previous studies show that the RTR policy and RTR promotion activities of the government significantly influence the decision making of families and the market environment (Qu et al., 2013). Researchers have also confirmed that the economic incentive measures adopted by the government can accelerate the achievement of the RTR goal. However, these measures also emphasize that if households are motivated to retrofit their toilets as a result of government initiatives, a further diffusion of RTR may fail as soon as the government program ends (Qu et al., 2013). Therefore, lessons should be learned from the experiences in RTR, and alternative RTR alternatives should be actively sought.

#### 3.1.4 From the perspective of grassroots promoters

The grassroots promoters of RTR, as represented by village heads or secretaries, can be found across all villages in Jiaozhou. Our survey data show that these promoters have more than 10 years of experience in serving villagers. Therefore, these promoters fully understand the actual situation of their villages, including the location of houses, population size, and families with financial difficulties. Specifically, they assist the members of the RTR government leading group in making the necessary investigations prior the RTR, implementing the retrofitting, and managing FS.

As shown in Fig. 3e, numerous grassroots promoters are involved in RTR as defined in the RTR policy documents issued by the government. The unequal distribution and unsustainable basic service systems across rural areas in Jiaozhou result from a combination of historical and current processes involving economic, infrastructural, and demographic factors, thereby creating a complex situation that is difficult to address (Cherunya et al., 2020). Therefore, grassroots promoters in each village face various challenges. To improve the accessibility of RTR information to farmers, grassroots promoters not only visit these farmers individually or in collaboration with the members of the RTR leadership group to convey the relevant RTR information but also adopt various methods (e.g., distributing brochures, hanging banners, and networking) to promote RTR, and thereby motivate these farmers to engage in this process (Xiao, 2019). Although subsidies are available, some poor families are unable to afford the remaining RTR costs. These families can also receive help from grassroots promoters to increase the number of village households with sanitary toilets.

Given that the political task of RTR has also attracted the attention of the government, grassroots promoters are often invited by local governments to participate in meetings about RTR. These meetings also serve as an information display and learning platform where grassroots promoters can meet and exchange experiences, such as in evaluating the quality and efficiency of RTR. All of the interviewed grassroots promoters agreed that they have obtained plenty of information from these meetings that helped improve the quality of retrofitted toilet, the motivation of farmers to engage in RTR, and the number of households with sanitary toilets.

Reward and punishment measures are also defined by the degree of attention given by the government. According to the provisions specified in the RTR policy document, if a village can complete the task within the specified time, then the associated grassroots promoters will be rewarded; otherwise, they will be criticized. All of these procedures positively contribute to improving the efficiency of RTR. Some researchers also highlight the importance of incentives in facilitating the construction of facilities for meeting the design standards and reducing FS pollution in the surrounding environment (Scott et al., 2013). However, in the actual RTR process, farmers are often regarded as passive recipients (Cherunya et al., 2020). Only through a strict supervision of the RTR process can the quality and efficiency of RTR be improved and a solid foundation for the long-term management of toilets can be laid out (Li, 2020).

#### 3.1.5 From the perspective of O&M personnel

The O&M system of RTR in Jiaozhou was founded under the jurisdiction of the government and property companies. O&M personnel are responsible for the extraction and transportation of FS and for repairing toilets based on information received from an intelligent platform (Cherunya et al., 2020). This intelligent platform not only shows the application requirements of farmers but also presents the work dynamics of the O&M team in real time (Schoebitz et al., 2017). A long-term O&M database are also established to scientifically guide FS management. The intelligent platform is presented in the supplementary material. Our survey data show that the employed O&M personnel have undergone professional trainings before becoming regular employees.

As shown in Fig. 3f, after households complete RTR, the functionality of toilets should be considered (Roubík et al., 2020). These toilets do not always work normally in the long term. Every household toilet needs regular maintenance, such as pumping FS once every two to four months to prevent overflow from the FS storage tank. The RTR policy document issued by the government provides many employment opportunities for local farmers, who can engage in toilet O&M to increase their family income. The workload of O&M personnel increases along with the number of households that apply for pumping FS or repairing toilets. Such increase in workload, in turn, increases the number of required O&M personnel. The efficiency of O&M personnel is affected by human, equipment, and environmental factors that either stimulate (i.e., the number and proficiency of O&M personnel and the number and performance of suction trucks) or suppress (i.e., distance) the satisfaction of farmers with sanitary toilets and their motivation to enegage in RTR. The newly recruited O&M personnel can receive skills training to improve their service proficiency. If the dispatch center receives complaints from farmers about poor O&M quality, then the O&M team will be criticized and undergo additional skills training to ensure service efficiency and quality. In terms of equipment, the FS suction truck (equipped with GPS) purchased for every 1500 persons can meet the current O&M needs. However, the negative result of O&M comes from the long distance for FS transportaiton. At the same time, when users applying for FS extraction or toilet repair reside in faraway locations, those users who are located closest to the O&M personnel will be prioritized.

#### 3.1.6 From the perspective of toilet supplier and construction teams

The diffusion of improved toilets presents a challenge because their successful adoption calls for a change in personal behavior, daily life, and perhaps even social norms (Ramani et al., 2012). To guarantee a successful adoption, the government not only introduced innovations in policy but also innovations in rural toilet subsidies. As shown in Fig. 3g, driven by RTR policies and rural market demand, many toilet suppliers provide high-quality toilet products to farmers in rural areas. Previous studies also show that farmers who face financial difficulties are having difficulty investing in RTR (Qu et al., 2013). By contrast, relatively young households generally belong to the upper–middle income group and are capable of investing in other toilet facilities if the government-subsidized ones are unsatisfactory. An increase in the number of toilet suppliers provides farmers with varies of toilet products and high-quality after-sales service that can improve their satisfaction and motivate them to engage in RTR. In addition, the market competition facilitates cooperation between the government and toilet suppliers in providing cheap and high-quality toilets. As a result, the number of households with sanitary toilets can be increased rapidly within a short time.

Survey results show that the number of toilet construction teams also increases as the number of households with sanitary toilets increases within a short time. Toilet construction teams face challenges in the RTR process, such as narrow village alleys, hard soil layers, and high surface water. To meet the standards specified in GB 19379–2012, these teams should undergo multiple trainings that improve not only the quantity but also the quality of toilet installation. Hence, the satisfaction of households can be enhanced further.

#### 3.1.7 From the perspective of FS end users

The rural toilet revolution aims to create rural sanitation infrastructure and public services that serve everyone and turn waste into value (Cheng et al., 2018). After the implementation of RTR in Jiaozhou, the garden space in rural living areas has increased. Some of these garden spaces can be as wide as 6.7 × 10^5^ m^2^. With cooperation with WWTPs, these gardens gradually form beautiful scenic spots (Zhu et al., 2020) that facilitate FS resource recovery. Using treated FS to irrigate gardens also contributes to the ecological cycle of nutrient elements in FS (Penn et al., 2018).

As shown in Fig. 3h, the number of gardens is mainly affected by policy factors as reflected in the investment in these gardens. The policy document issued for RTR has increased the government investments in these gardens, and the increase in the number of sanitary toilets and WWTPs provides a sufficient amount of fertilizers for these gardens. A strong linkage between WWTPs and gardens is imperative to ensure sustainable service value growth in gardens. Increasing the number of FS-irrigated gardens, the value of garden byproducts, and the capacity of water conservation (Tamang et al., 2019) will also gradually increase the amount of carbon fixation and oxygen release (Tamang et al., 2019), the amount of nutrient cycling (Getahun et al., 2020; Gong et al., 2019; Penn et al., 2018), the value of purified air (Shao et al., 2019), and the value of soil conservation. Some grown trees in the garden can also provide people with important living and production materials, including food, fiber bioenergy, and pharmaceuticals (Xia et al., 2020), all of which have certain economic benefits.

However, the tree-type structure of rural gardens in Jiaozhou should be further optimized. These gardens are mainly constructed through the cultivation of tree seedlings and have a relatively single ecosystem that is not conducive to a balanced development of the value of garden ecological services (Baumgärtner and Bieri, 2006). Moreover, the trees in these gardens have a relatively slow growth rate at the early stage and require a relatively long time before they can produce ecological and economic benefits. Therefore, Jiaozhou should further clarify the short- and long-term development strategies of their rural gardens in order for their RTR and FS resources recovery initiatives to lead to a sustainable development.

### 3.2 Lessons learned from the Jiaozhou RTR

System dynamics are considered as simulation “sites” that can produce key insights into the ways and means through which RTR operates and changes over time. Robust support policies and subsidies are the most fundamental promoters of RTR in Jiaozhou. The rural toilet revolution in Jiaozhou has eventually realized a safe FS collection, storage, transportation, treatment, disposal, and resource recovery. Regions in developing countries with strong economic power can refer to the RTR model of Jiaozhou to improve their rural sanitation systems. Apart from constructing sanitary toilets based on local conditions, the government should also take the long-term O&M and FS resource recovery into consideration.

However, the CLD analysis reveals several limitations that hinder the popularization of RTR in Jiaozhou. For instance, if the government neither establishes a government-dominated top–down RTR support system nor provides sufficient financial subsidies, then the RTR model of Jiaozhou will lose its power source and lead to failure.

Meanwhile, for developing countries where the economy is lagging, rural infrastructure faces obstacle in terms of fund shortage. On one hand, the government investment in rural development is generally insufficient. The lack of investments in public infrastructure often limits productivity in rural areas. On the other hand, there is regional inequality in infrastructure investment in rural areas. Although the implementation of rural policies successfully promotes economic and social development, rural development continues to face insufficient funding for public facilities (including sanitation facilities), such as in the rural areas of Western China (Zhang et al., 2020). To increase the penetration rate of rural sanitary toilets, local governments can provide preferential policies to toilet enterprises and property companies to stimulate the internal vitality of the toilet market environment. The goal of the toilet revolution can only be achieved by launching a market-oriented promotion of RTR.

## 4 Conclusions

The main stakeholders in RTR include household users, WWTPs, local governments, grassroots promoters, O&M personnel, toilet supplier and construction teams, and FS end users. Given that the complexity of RTR involving different stakeholders in China has never been described in the literature, this paper takes the RTR in Jiaozhou as an example and then identifies and establishes causal relationships among its major stakeholders and factors based on feedback.

Given a relatively backward rural infrastructure, this study highlights the importance of clarifying the relationship between the main stakeholders and the rural toilet market, especially the role of the main stakeholders in the implementation of the RTR process, the impact of policies, subsidies, and technologies, and the satisfaction and cooperation of stakeholders in RTR. The feedback and mutual influence information obtained from the CLD of these major stakeholders can help optimize the multi-element and multi-mode system of RTR. The findings of this research can provide scientific reference and suggestions to ensure an efficient RTR in developing countries.

## Declaration of competing Interest

The authors declare that they have no known competing financial interests or personal relationships that could have appeared to influence the work reported in this paper.
