# Supplementary material for: Using system dynamics to assess the complexity of rural toilet retrofitting: Case study in eastern China
Source: J Environ Manage. 2021 Feb 15;280:111655. doi: 10.1016/j.jenvman.2020.111655 (PMC7816123; doi:10.1016/j.jenvman.2020.111655)
Supplement: Multimedia component 1 [file mmc1.docx]

Using system dynamics to assess the complexity of rural toilet retrofitting: Case study in Eastern China

**Supplementary material**


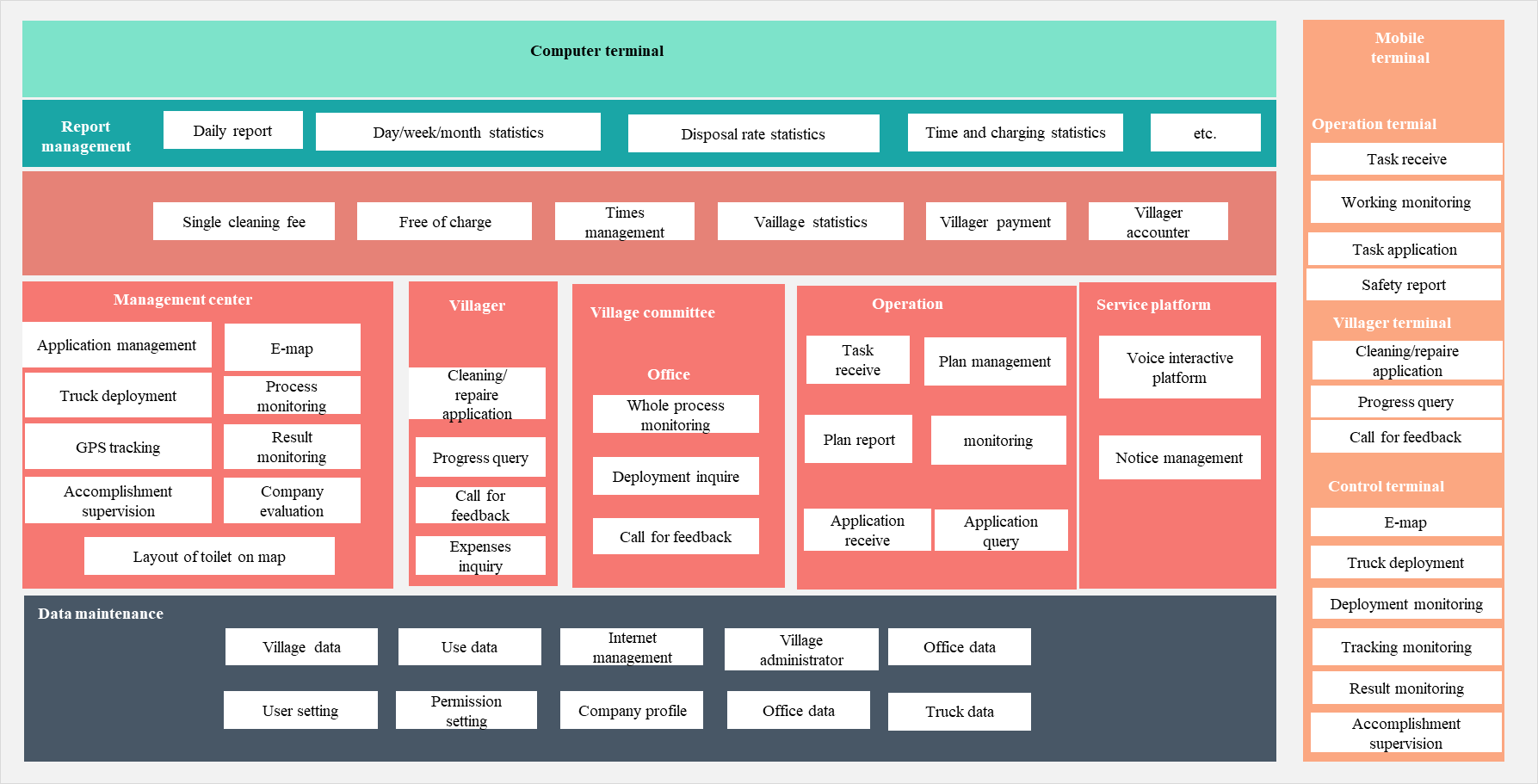


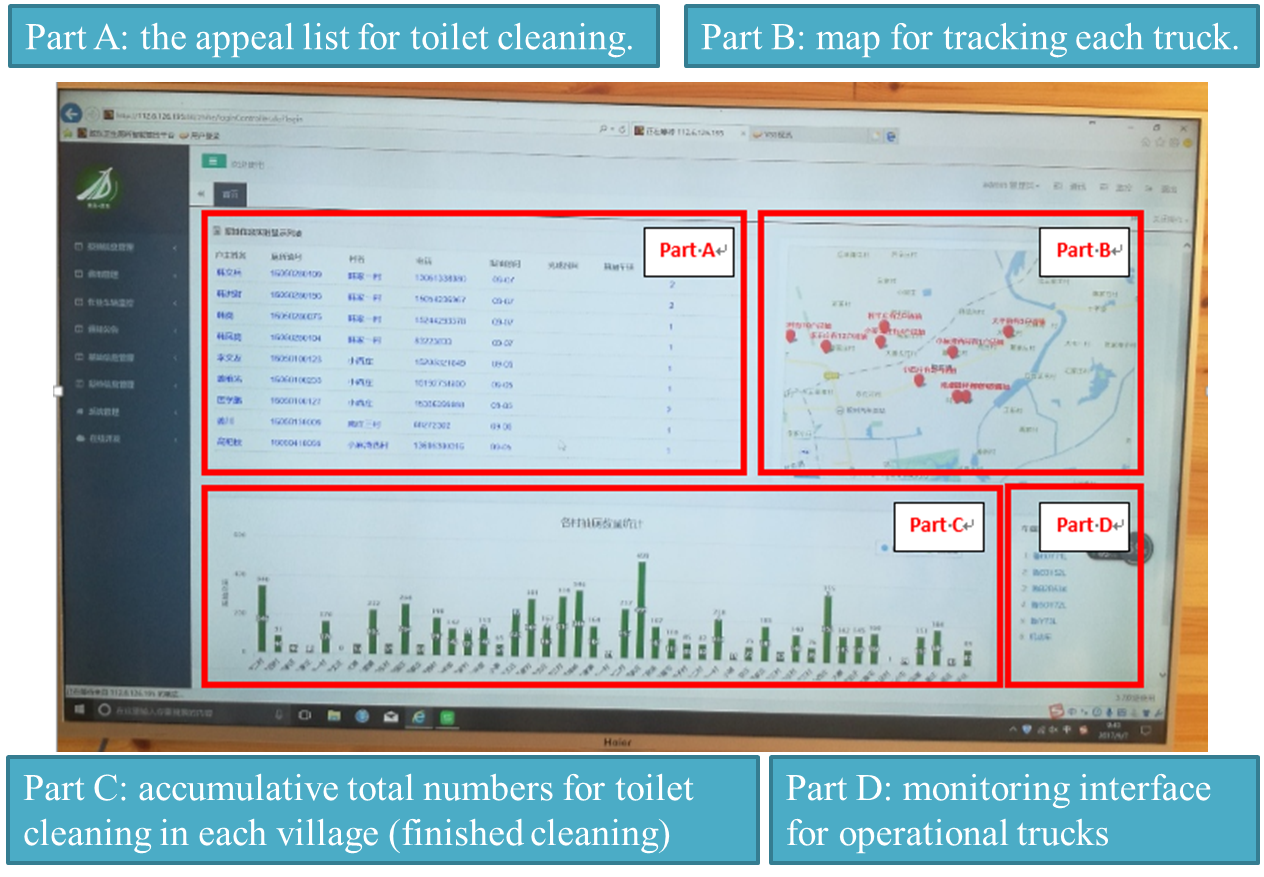


Fig. S1. Intelligent O&M platform for toilet retrofitting in rural Jiaozhou


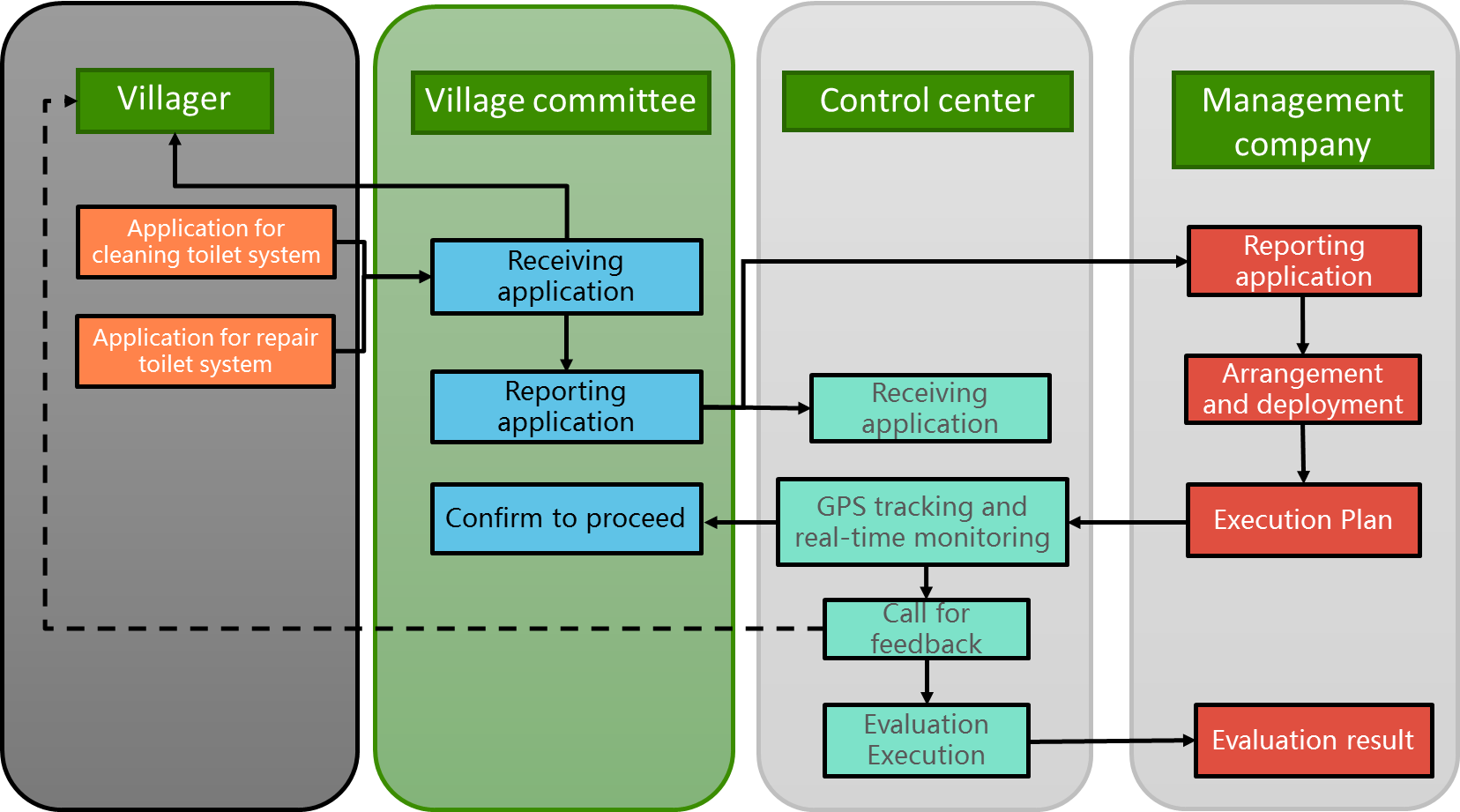


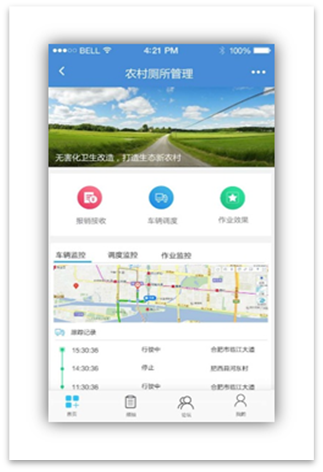

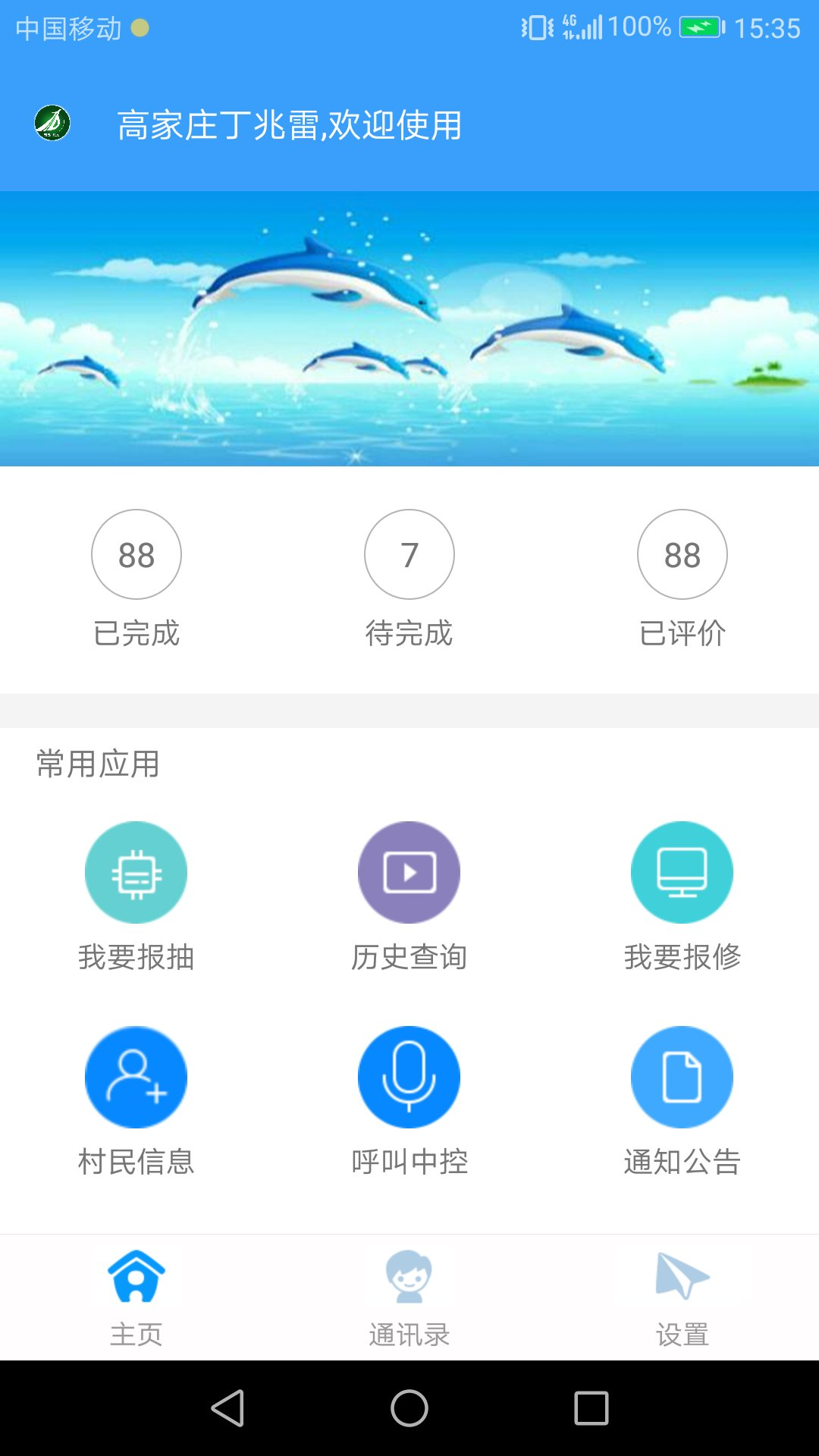


Fig. S2. FS management platform and village terminal APP in mobile


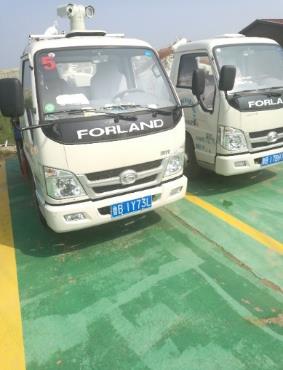


Fig. S3. FS suction truck (equipped with GPS, real-time driving records are presented on the intelligent platform)


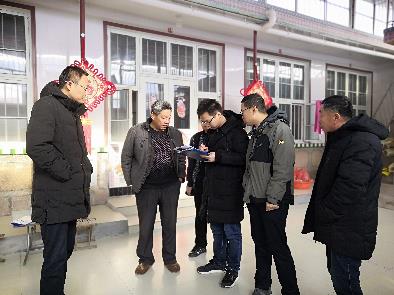

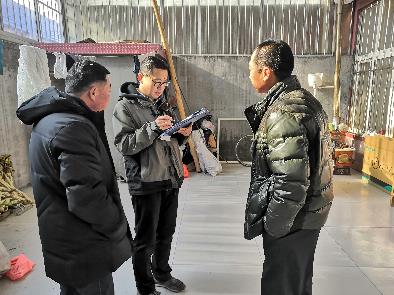


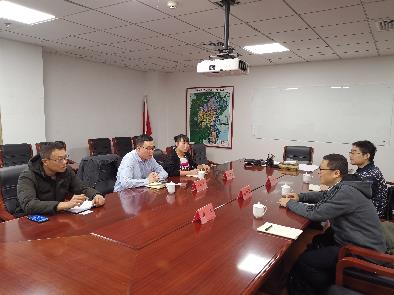

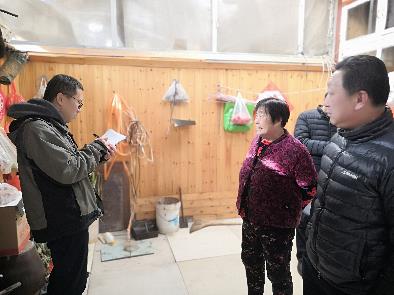


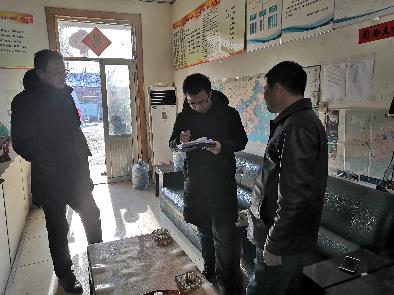

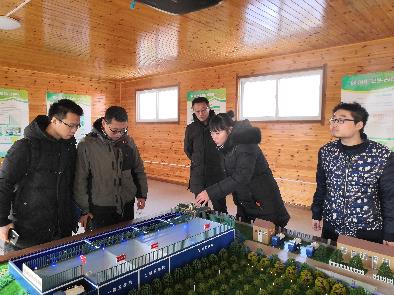


Fig. S4. Questionnaire survey and semi-structured interview


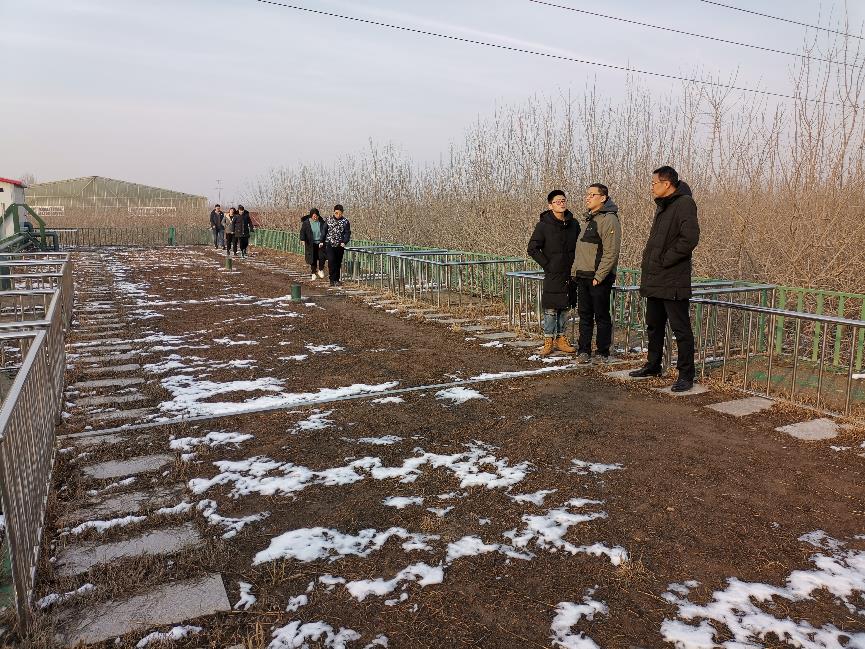


Fig. S5. Village-level WWTP without pipeline system (collected FS is transported here for treatment and the effluent is used for irrigation in the garden)

| 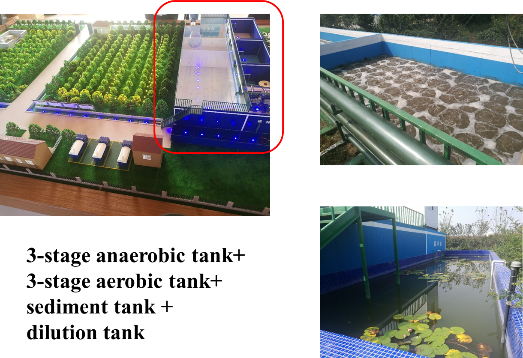 |
| --- |

Fig. S6. A model of village-level WWTP (red color box) with garden nearby, the aerobic tank with aeration and the sediment tank


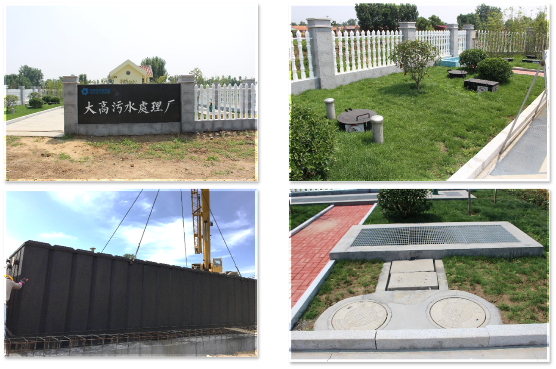


Fig. S7. A town-level WWTP with local pipeline system (all kinds of wastewater from household are transported here via local pipeline system, most units are built underground)
